# Spectrum of ascending aortic aneurysms at a peri-urban tertiary hospital: an echocardiography-based study

**DOI:** 10.3389/fcvm.2023.1209969

**Published:** 2023-07-10

**Authors:** Ruchika Meel, Michael Hasenkam, Ricardo Goncalves, Kelly Blair, Shungu Mogaladi

**Affiliations:** ^1^ Division of Cardiothoracic Surgery, Faculty of Health Sciences, University of the Witwatersrand, Johannesburg, South Africa; ^2^ Aarhus University Hospital, Aarhus, Denmark; ^3^ Life The Glynnwood Hospital, Johannesburg, South Africa; ^4^ Chris Hani Baragwanath Academic Hospital, Johannesburg, South Africa; ^5^ Division of Cardiothoracic Surgery, Department of General Surgery, Charlotte Maxeke Hospital and University of the Witwatersrand, Johannesburg, South Africa

**Keywords:** Africa, aneurysms, echocardiography, strain imaging, ascending aorta, aortic regurgitation 2

## Abstract

**Introduction:**

Thoracic ascending aortic (TAA) aneurysms are an important cause of disability and death and require early detection for effective management. Currently, there is a paucity of data from Africa pertaining to TAA aneurysms. This study describes the spectrum of TAA aneurysms at a peri-urban tertiary hospital.

**Methods:**

A descriptive retrospective study based on clinical and echocardiographic imaging data of patients with TAA aneurysms from October 2017–October 2022. Advanced strain imaging was performed to measure left ventricular (LV) basal, apical, and global longitudinal strain as well as circumferential strain (CS) of the ascending aorta as a proxy measurement of aortic compliance.

**Results:**

The study comprised 139 cases of TAA aneurysms (52.5% females) with a mean age of 50 ± 14.8 years with 45 age and gender matched controls. Most cases (95%) were of African ethnicity. The main etiologies were hypertension (41.7%), HIV (36.6%), connective tissue disease (10.7%), congenital (2.2%) and mixed pathologies (8.6%). Two-thirds of patients (69.7%) presented in heart failure, 10% presented with aortic dissection. Thirty percent of the patients were classified as New York Heart Association (NYHA) class I, 59.7% NYHA II, 8.6% NYHA III and two patients NYHA class IV. Echocardiography revealed enlarged aortic dimensions compared to controls (*P* < 0.001). TAA aneurysms were complicated by severe aortic regurgitation (AR) in half (50.3%) of patients, moderate AR in 25.8%, and mild AR in 14.3%. The mean LV ejection fraction (46.9 ± 12.7%) was reduced compared to controls (*P* < 0.001). Aortic CS was reduced compared to controls [4.4 (3.2–6.2) % vs. 9.0 (7.1–13.4) %, *P* < 0.001]. Aortic stiffness was higher in the aortic aneurysm group compared to controls (15.39 ± 20.65 vs. 5.04 ± 2.09, *P* = 0.001). LV longitudinal strain (−13.9 ± 3.9% vs. 18.1 ± 6.7%), basal CS (−13.9 ± 5.6% vs. −17.9 ± 5.8%) and apical CS (−8.7 ± 8.5% vs. −30.6 ± 3.8%) were reduced compared to controls (*P* < 0.001). Most patients were on diuretic and anti-remodeling therapy. Surgery was performed in 29.4% and overall mortality was 7.9%. Mortality for acute aortic dissection was 40%.

**Conclusion:**

TAA aneurysms associated with hypertension and HIV are common in this predominantly African female population and are associated with considerable morbidity and mortality. Two-dimensional echocardiography and advanced strain imaging are potential tools for detecting and risk stratifying TAA aneurysms.

## 1. Introduction

Aneurysms of the ascending aorta often develop undetected due to their non-specific and late-stage clinical presentation. These aneurysms can rapidly lead to death due to aortic rupture or dissection (1) and thus are an important cause of mortality in adults. Although no specific data exists regarding the true mortality from thoracic ascending aortic (TAA) aneurysms, studies have shown a prevalence of aortic aneurysms ranging from 1.6% to 7.2% in a general population aged 60 years or older (2). Mortality due to aortic aneurysms is estimated to be between 157,357 and 18,899 (3) globally according to the global burden of disease (GBD) 2019, thus making death due to aortic aneurysms a significant public health concern.

TAA aneurysms occur more commonly in males than females with a ratio of 1.7–3:1 and with a mean age of 65 years at presentation, however this distribution may change with age. Ogeng'o et al. in their paper in Kenya showed a male-to-female ratio of 1.4:1 in patients before the age of 40 years, however a 1:1 ratio before 50 years and a 1:2, male-to-female ratio after 50 years of age (4).

In terms of the main aetiologies causing TAA aneurysms, it is well documented that atherosclerosis, hypertension, connective tissue and inflammatory disease, congenital heart disease, and infectious causes such as syphilis and human immunodeficiency virus (HIV) can cause TAA aneurysms (5). Further to this, ethnicity, geography, and burden of risk factors can also dictate the pattern of aneurysmal disease of the aorta (4). In African populations, hypertension and syphilis have been noted as frequent risk factors whereas the most frequent risk factors in people of European-origin are hypertension, ischemic heart disease (IHD) and age (6, 7).

Recent work by Høgh et al. has shown a direct association between HIV and TAA aneurysms. It was found that there was a four-fold higher odds of aortic aneurysms compared to uninfected controls likely due to the proinflammatory state associated with HIV (8, 9). Given that Southern Africa has amongst the highest rates of people living with HIV globally, HIV is thus an important cause of aortic aneurysms in this population (10, 11).

Strain imaging of the aorta using speckle tracking echocardiography (STE) has emerged as an important feasible and reproducible bedside tool to assess aortic stiffness (12). In the context of proximal TAA aneurysms use of circumferential strain may impact the prognosis of these patients by aiding risk stratification of patients that are at risk for further aortic dilatation or dissection (13, 14).

As it stands, there is a paucity of data on the clinical spectrum, screening processes and management of TAA aneurysms in Africa. As a result, this study aims to begin building information around demographics, clinical characteristics, two-dimensional echocardiography and strain imaging of TAA aneurysms, and morbidity and mortality by characterizing the spectrum of ascending aortic aneurysm in a peri-urban tertiary hospital in South Africa.

## 2. Methods

### 2.1. Study population

This was a descriptive retrospective cross-sectional study of 139 patients with confirmed TAA aneurysms seen at Chris Hani Baragwanath Academic Hospital (2017–2022). The data were extracted from an established database of patients with aortopathy as well as from pre-recorded echocardiographic images and surgical presentation reports. Results of routinely performed blood tests for clinical management of patients were also retrieved and analysed.

Data of forty-five age and gender matched healthy controls were extracted from a prior study that included normal participants (M200977). The inclusion criteria were (i) age greater than 18 years, (ii) confirmed TAA aneurysm (aorta >40 mm in size or dilation greater than 1.5 times the normal diameter of the adjacent healthy arterial segment) (15–17). The exclusion criteria were (i) suboptimal image quality of aorta (ii) isolated abdominal aortic or arch aneurysms (iii) extensive missing data. Eight patients were excluded due to extremely poor echocardiographic imaging quality, five due to extensive missing data, one due to an isolated arch aneurysm and two due to an exclusive abdominal aortic aneurysm.

The study was conducted in accordance with the Declaration of Helsinki (as revised in 2013) available at: https://www.wma.net/wp-content/uploads/2016/11/DoH-Oct2013-JAMA.pdf. Ethics approval for the study was obtained from the University of the Witwatersrand ethics committee (M170389).

### 2.2. Echocardiographic examination

Aortic measurements were obtained as per the 2015 American Society of Echocardiography guidelines using a Philips EPIQ 9 system (18). Circumferential Strain (CS) of the ascending aorta (AAO) was measured using Philips QLAB version 11.0 software for the left ventricle which allowed offline semi-automated analysis of speckle-based strain two-dimensional speckle-tracking software (Amsterdam, Netherlands). All echocardiographic measurements were performed by an experienced cardiologist and clinical technologist.

Transthoracic echocardiographic examinations were performed on all patients in the left lateral position. An S5-1 transducer on a Philips EPIQ 9 system was used to obtain the aortic measurements from parasternal long axis views where the aortic root and proximal aorta and the left ventricle (LV) could be visualized. Measurements at four different levels in the proximal aorta were made namely (i) the aortic annulus (AA); (ii) sinuses of Valsalva (SOV); (iii) Sino-tubular junction (STJ); and (iv) the proximal ascending aorta (AAO). From the same window, with appropriate probe rotation, two-dimensional short-axis views at the level of the aortic valve plane were acquired and the image depth and the sector width were adjusted to optimize proximal aorta visualization. Zoomed-in images of both left ventricle outflow tract (LVOT) in the parasternal long-axis view and of the aortic valve in the parasternal short-axis view were obtained and recorded.

As recommended by the 2015 American Society of Echocardiography (ASE) Guidelines, the aortic annulus was measured at mid-systole from the inner-edge to inner-edge. All other aortic root measurements (i.e., maximal diameter of the sinuses of Valsalva (SV), the Sino-tubular junction (STJ), and the proximal ascending aorta (AAO) were made at end-diastole (QRS complex onset), in a leading-edge-to-leading-edge convention (18). The European Society of Cardiology (ESC) 2017 valvular heart disease guidelines were used to quantify the severity of valvular regurgitation (19). The measurements of LV diastolic function were performed following the standard guidelines on diastolic function by Nagueh et al. (20). Measurements relating to the right ventricle were based on the ASE guidelines (21).

Two-dimensional (2D) speckle-tracking (ST) echocardiography was used to determine the circumferential strain (CS) (22). Images of the ascending aorta were first obtained in the high parasternal long-axis view until the largest diameter of the ascending aorta was visualized. Then, akin to the measurement of CS of the left ventricle, in short axis view a loop was manually drawn along the inner edge of the aortic wall during systole and then an additional loop near the outer edge of the aortic wall was automatically generated by the software. The software then divided the aortic wall image into six equally sized segments and the global circumferential ascending aortic strain was calculated as the mean value of the peak CS of the six segments. The data was then transferred and analysed offline using the Philips X-Celera workstation.

Aortic stiffness index (*β*_2_) was calculated as follows (23):

$$\beta_{2}=\frac{\ln\left(\frac{SBP}{DBP}\right)}{Aortic\;CS}$$

***β*_2_:** Aortic Stiffness index

***SBP*:** Systolic Blood Pressure

***DBP*:** Diastolic Blood Pressure

***CS*:** Circumferential strain

Two-dimensional echocardiographic images were obtained at end-expiration from LV apical long-axis 4-, 3-, and 2-chamber views with frame rates between 60 and 80 frames per second (24). Three consecutive cardiac cycles were recorded and averaged. Global LV systolic strain was calculated by averaging the three apical views as previously described.

### 2.3. Statistical analysis

All computations for this data were carried out using Statistica version 14.00.15. Continuous variables were expressed as mean and standard deviation (SD) or median (interquartile range). Student's *t*-test or Mann-Whitney *U* test were used to compare continuous variables. Categorical data were expressed as percentages. Categorical variables were evaluated by the Chi-square test. Pearson's correlation coefficient was used to measure the association between two continuous variables. A *P* value of ≤0.05 was considered statistically significant. One-way analysis of variance (ANOVA) was used to compare continuous variables and *post hoc* comparisons were performed using Scheffé test. For Non-parametric data Kruskal-wallis test was performed with *post hoc* testing using Dunn's test.

## 3. Results

The demographic and clinical characteristics of study patients are summarised in Table 1. Most of the patients were female and of African ethnicity (95%), six patients were of mixed race and one patient was of Indian descent. There was only one patient of European origin. This patient presented with acute aortic dissection and demised soon after admission, and so was excluded from the current study due to lack of data.

**Table 1:** Clinical characteristics of the study population and control group.

Variable	Study patients	Controls	*P*-value
	*N* = 139	*N* = 45	
Age (years)	50 ± 14.8	46.3 ± 6.7	0.106
Gender (M/F)	66/73 (47.4%/52.6%)	24/21 (53.3%/47.7%)	0.9
BSA (m^2^)	1.73 ± 0.2	1.81 ± 0.2	0.02
SBP (mmHg)	137.2 ± 22.1	128.4 ± 10.5	0.01
DBP (mmHg)	72.7 ± 16.6	81.6 ± 10.2	<0.001
Heart rate (bpm)	80.6 ± 14.7	73.7 ± 10.7	0.004
NYHA class (I/II/III/IV)	(30.2%/59.7%/8.6%/1.4%)		
Co-morbidities			
Hypertension	58 (41.7%)		
HIV	51 (36.7%)		
Diabetes	4 (2.8%)		
Medication			
Carvedilol	62 (44.6%)		
Enalapril	87 (62.5%)		
Coversyl	6 (4.3%)		
Furosemide	84 (60.4%)		
Hydrochlorothiazide	15 (10.7%)		
Amlodipine	21 (15.1%)		
Nifedipine XL	10 (7.2%)		

BSA, body surface area; DBP, diastolic blood pressure; SBP, systolic blood pressure; NYHA, New York Heart Association.

Regarding the presenting clinical symptoms, dyspnoea as a result of heart failure (70%) was the most common complaint. Fourteen (10%) patients presented with chronic aortic dissection. Five patients presented with acute aortic dissection but were excluded from the study due to a lack of data.

In terms of medical management, diuretic and angiotensin-converting enzyme inhibitor use for heart failure was common. However, Beta-blocker use was comparatively restricted likely due to fear of worsening aortic regurgitation related to prolonged diastole from bradycardia. All patients with HIV were on anti-retroviral medication.

### 3.1. Aetiological distribution in the study population

**Figure 1:**
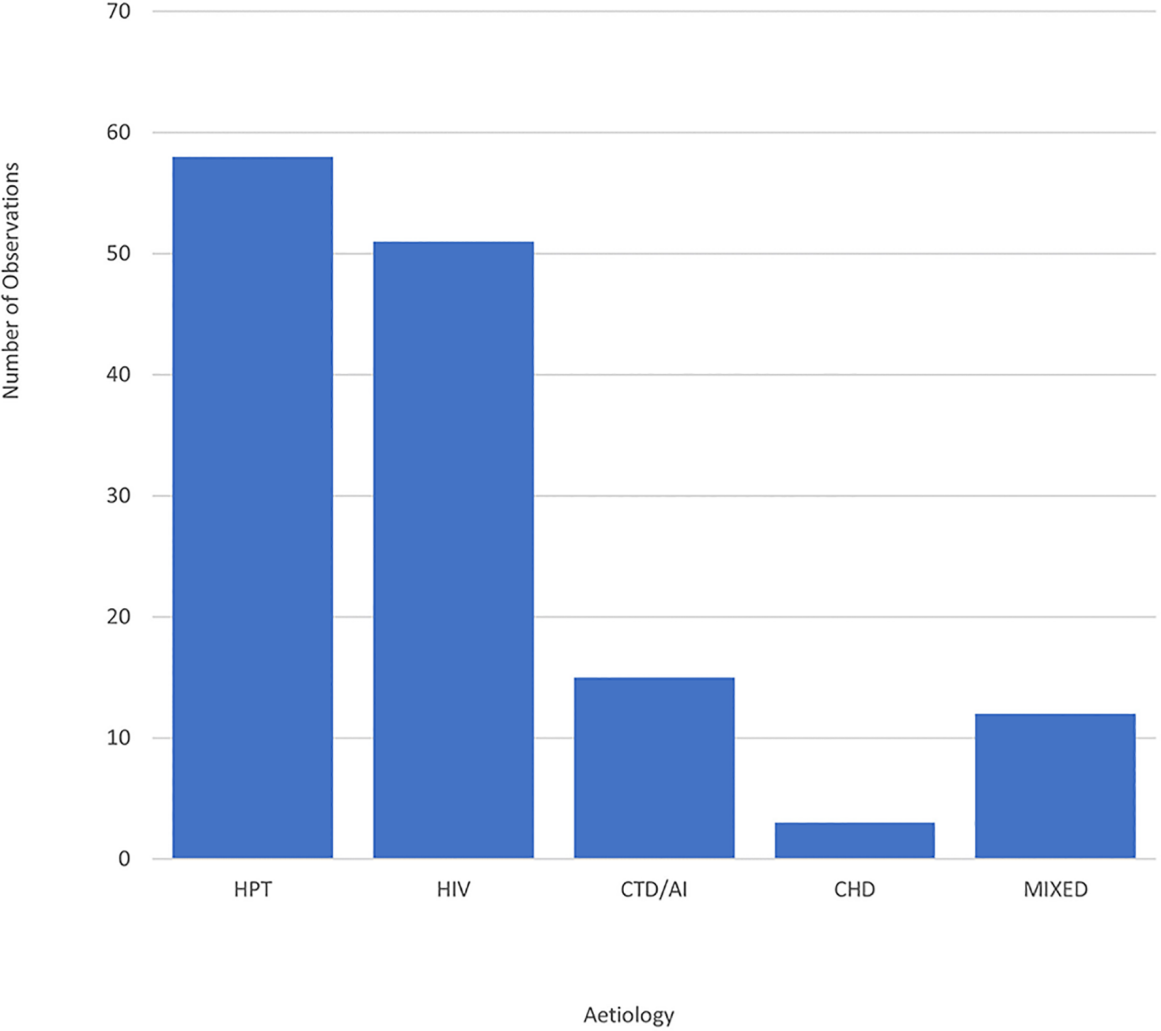
Bar graph showing the distribution of TAA aneurysm cases by aetiology. CHD, congenital heart disease; CTD/AI, connective tissue disease/autoimmune; HIV, human immunodeficiency virus; HPT, hypertension; Mixed, mixed aetiologies.

The main aetiologies of TAA aneurysms are depicted in Figure 1. Hypertension and HIV were the most common aetiologies. There were eight cases of Marfan syndrome, two cases of bicuspid aortic valve, two non-specified cases of connective tissue disease and one case of Ehlers-Danlos syndrome, who presented with pulmonary embolism. In terms of autoimmune aetiologies, there were two cases of Takayasu's arteritis. Regarding cases with congenital heart disease, there was a participant who had a congenital repaired ventricular septal defect (VSD) with subaortic membrane and right ventricular outflow tract obstruction, another patient with a restrictive VSD, as well as one case of Tetralogy of Fallot with a concurrent TAA aneurysm.

In 8.6% of the cases there were multiple or mixed aetiologies. There were two participants who were HIV positive and had a congenital heart lesion. One of these patients had a VSD and the other had a VSD combined with a subaortic membrane. There were also two cases of TAA aneurysms in patients who had had coronavirus disease-associated pneumonia, one of these cases had a background of Marfan syndrome and the other had a background of hypertension. One patient with HIV had a history of pulmonary tuberculosis. There were three cases that had dyslipidaemia, one patient was also HIV positive, one had hypertension and ischaemic heart disease and the third patient had hypertension with diabetes, all three were on lipid-lowering therapy.

Based on the Treponema Pallidum Haemagglutination test, syphilis was suspected in four cases. Two of which had HIV and two were hypertensive. Eleven cases of TAA aneurysms had concurrent arch or descending aorta aneurysms. Of these, seven had HIV, one had Takayasu's arteritis and three were hypertensive.

In terms of differences based on gender, there was no statistical difference between genders in terms of the main aetiologies of hypertension (27 M vs. 38 F, *P* = 0.42) and HIV (28 M vs. 35 F, *P* = 0.68).

### 3.2. Echocardiographic characteristics of the study population

**Figure 2:**
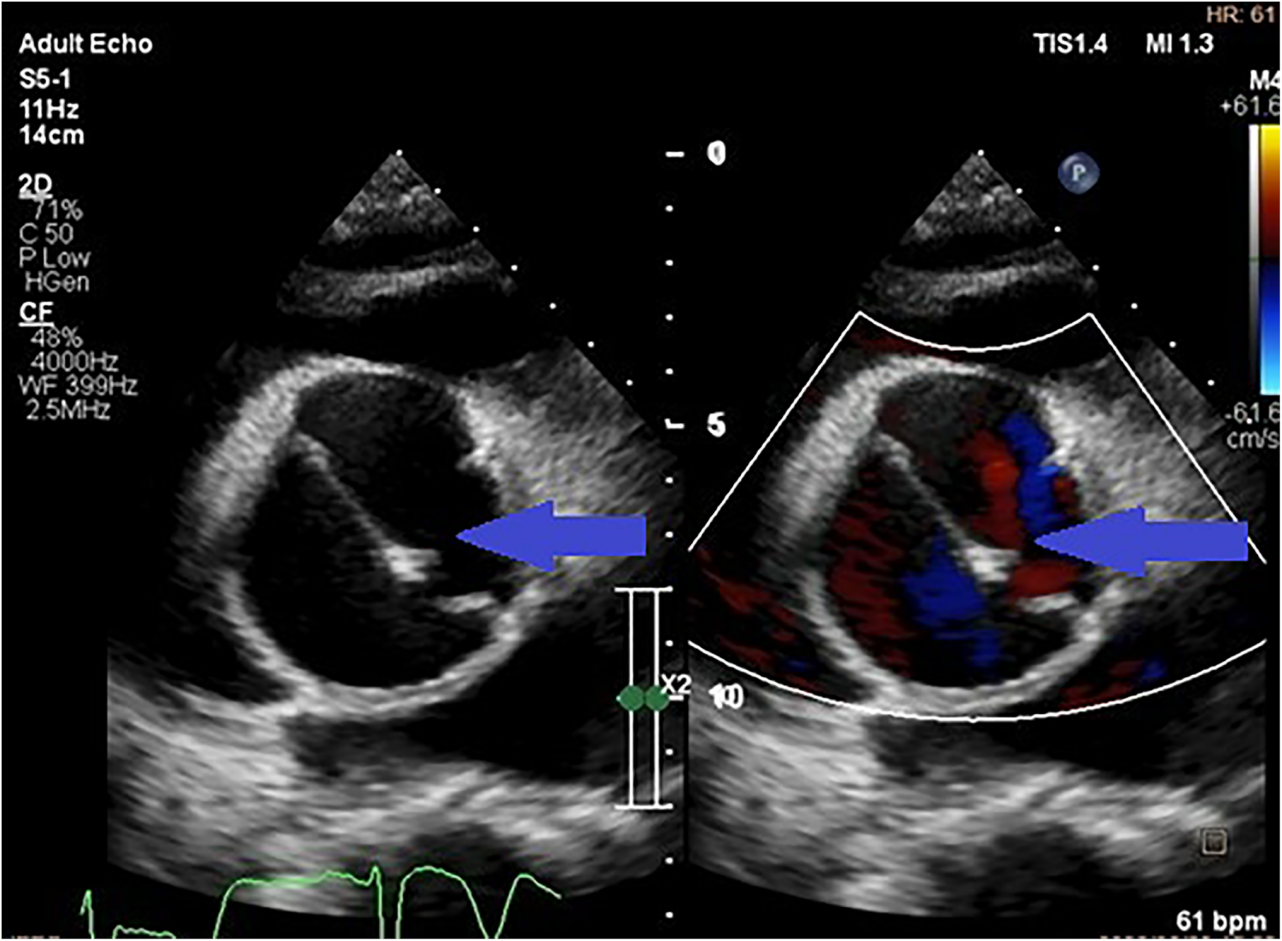
Parasternal short axis transthoracic view showing a dissection flap in an aneurysmal thoracic aorta (indicated by the blue arrows) in a patient with uncontrolled hypertension.

The aorta was significantly enlarged from the root to the ascending aorta compared to controls. Patients that presented with aortic dissection had larger diameters compared to the group without dissection (54.5 ± 8.0 mm vs. 48.2 ± 10.3 mm, *P* = 0.02). These results remained statistically significant when indexed to body surface area (36.3 ± 11.5 mm/m^2^ vs. 28.2 ± 7 mm/m^2^, *P* < 0.001). Nine patients with dissection were hypertensive (Figure 2), four had HIV and one had connective tissue disease (Table 2).

**Table 2:** Echocardiographic characteristics of the study population compared to control group.

Variable	Study patients (*n* = 139)	Controls (*n* = 45)	*P*-value
Left ventricular function			
LV EDD (mm)	55.2 ± 10.1	42.7 ± 4.7	<0.001
LV EDD (mm/m^2^)	32.6 ± 7.4	23.6 ± 3.4	<0.001
LV ESD (mm)	41.8 ± 11.0	29.1 ± 4.9	<0.001
LV ESD (mm/m^2^)	29.4 ± 7.4	16.1 ± 2.7	<0.001
IVSD (mm)	10.32 ± 2.29	7.02 ± 1.52	<0.001
IVSD (mm/m^2^)	6.05 ± 1.53	4.38 ± 1.09	<0.001
LVPWD (mm)	10.32 ± 2.29	7.34 ± 1.36	<0.001
LVPWD (mm/m^2^)	6.05 ± 1.53	4.59 ± 1.07	<0.001
LV mass (g)	230.76 ± 89.62	96.87 ± 38.77	<0.001
LV mass (g/m^2^)	136.42 ± 53.90	60.16 ± 24.22	<0.001
EDV (ml)	162.7 ± 73.1	84.4 ± 19.5	<0.001
EDV (ml/m^2^)	96.3 ± 44.2	46.3 ± 10.6	<0.001
ESV	89.9 ± 54.8	33.1 ± 15.1	<0.001
ESV (ml/m^2^)	53.2 ± 32.8	17.9 ± 7.1	<0.001
LVEF (%)	46.7 ± 12.7	63.3 ± 7.0	<0.001
E wave (cm/s)	78.3 ± 30.2	77.7 ± 18.6	0.910
E/A ratio	69.7 ± 27.4	59.9 ± 15.8	0.020
E’ lateral (cm/s)	8.0 ± 3.8	11.9 ± 2.9	<0.001
Lateral E/E’ (cm/s)	9.6 (6.6–14.2)	6 (5.2–8.2)	0.002
S’ lateral (cm/s)	6.82 ± 2.1	8.3 ± 1.9	<0.001
Left atrial volume (ml)	58.5 ± 25.6	41.6 ± 14.7	<0.001
Left atrial volume (ml/m^2^)	34.4 ± 15.2	23.3 ± 7.4	<0.001
Aortic measurements			
Annulus (mm)	23.5 ± 4.3	19.6 ± 2.5	<0.001
Annulus (mm/m^2^)	13.8 ± 2.9	10.8 ± 1.4	<0.001
Sinuses (mm)	44.1 ± 9.2	28.8 ± 7.9	<0.001
Sinuses (mm/m^2^)	26 ± 6.5	15.7 ± 3.5	<0.001
Sino-tubular junction (mm)	46.2 ± 10.1	26.0 ± 3.2	<0.001
Sino-tubular junction (mm/m^2^)	27.3 ± 7.4	14.3 ± 1.6	<0.001
Ascending aorta (mm)	48.9 ± 10.1	26.5 ± 3.0	<0.001
Ascending aorta (mm/m^2^)	28.9 ± 7.8	14.5 ± 1.6	<0.001
Valvular pathology			
Aortic regurgitation (none/mild/moderate/severe) (%)	9.3/14.3/25.8/50.3	-	
Mitral regurgitation (none/mild/mod/severe) (%)	52.5/33.1/10.8/3.6	-	
Tricuspid regurgitation (none/mild/mod/severe) (%)	43.8/41.7/7.2/7.2	-	
Right ventricle function			
Right ventricle base (mm)	34.7 ± 8.2	29.5 ± 5.5	<0.001
Right ventricle base (mm/m^2^)	20.2 ± 5.4	16.3 ± 3.3	<0.001
TAPSE (mm)	19.9 ± 11.1	18.6 ± 4.5	0.460
S’ (cm/s)	11.12 ± 3.2	11.2 ± 2.2	0.840
E’ (cm/s)	8.9 ± 3.6	10.5 ± 3.1	0.008
A’ (cm/s)	12.6 ± 7.5	11.6 ± 3.0	0.390
E’/A’ ratio	0.82 ± 0.6	0.97 ± 0.3	0.170
PASP (mmHg)^a^	30.7 ± 20.8	17.6 ± 5.7	<0.001
Strain parameters			
Left ventricle GLS (%)	−13.9 ± 3.9	−18.1 ± 6.7	<0.001
Left ventricle basal CS strain (%)	−13.9 ± 5.6	−17.9 ± 5.8	<0.001
Left ventricle apical CS strain (%)	−19 (−23 to −12.7)	−24.6 (−32.7 to −20.6)	0.002
Aortic CS (%)^b^	4.4 (3.2–6.2)	9.0 (7.1–13.4)	<0.001
Aortic stiffness index (*β*_2_)	15.39 ± 20.65	5.04 ± 2.09	0.001

Data are presented as mean ± SD, median (IQR) or %. CS, circumferential strain; EDV, end-diastolic volume indexed; ESV, end-systolic volume indexed; GLS, global longitudinal strain; IVSD, interventricular septum diameter; LV, left ventricle; LV EDD, left ventricular end-diastolic diameter; LVEF, left ventricular ejection fraction; LVESD, left ventricular end-systolic diameter; LVPWD, left ventricular posterior wall diameter; PASP, pulmonary artery systolic pressure; TAPSE, tricuspid annular plane systolic excursion.

^a^ PASP measurement was feasible in 32 controls and 108 study patients.

^b^ Aortic CS and *β*_2_ was not feasible in 14 patients that presented with aortic dissection of the ascending aortic aneurysm due to intimal disruption **IVSD in diastole.

**Figure 3:**
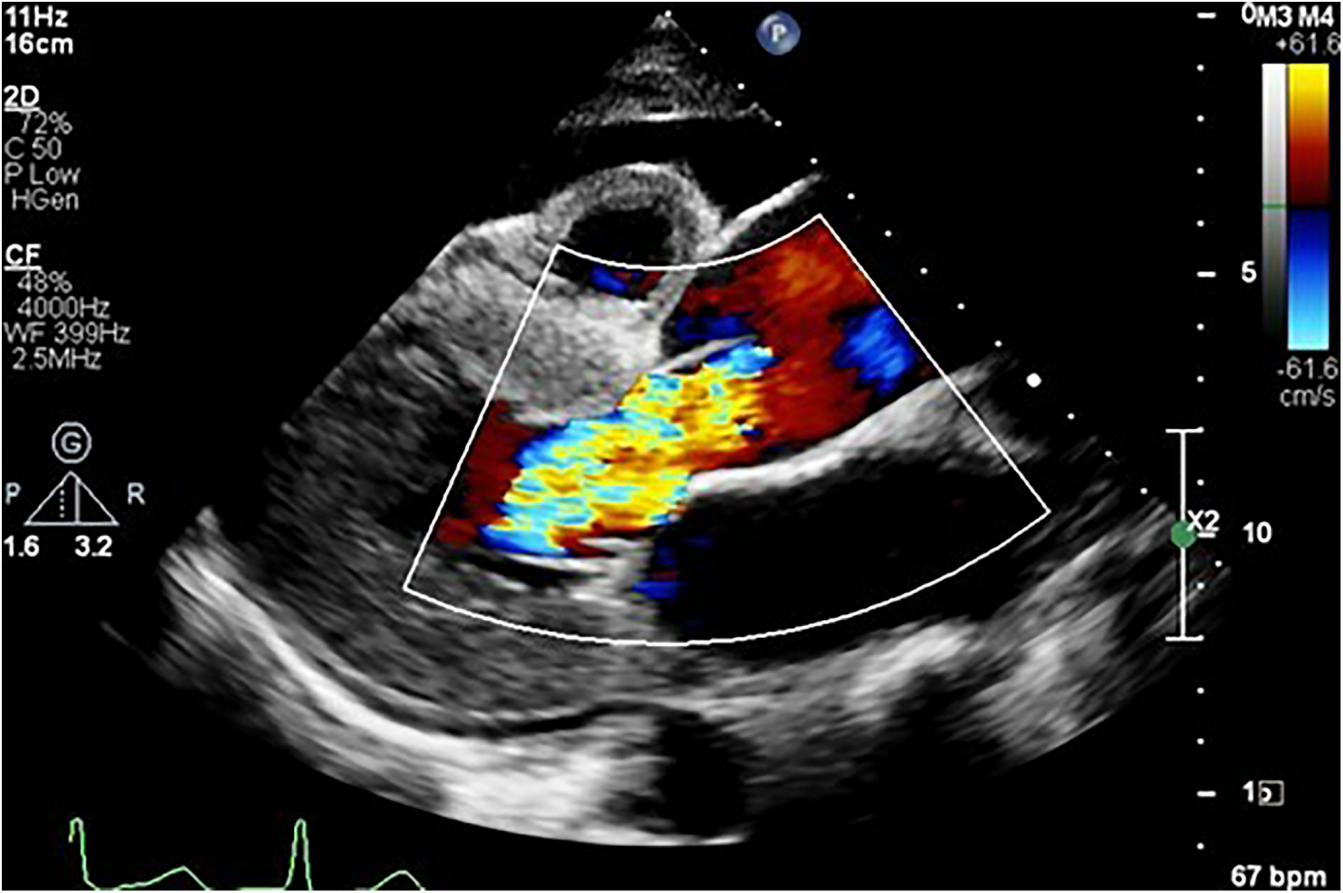
Parasternal long axis view showing an aortic aneurysm secondary to hypertension complicated by heart failure, as evidenced by the anterior pericardial effusion secondary to severe aortic regurgitation.

Compared to controls, patients with aortopathy had a significantly dysfunctional LV. As most patients presented in decompensated heart failure, secondary to aortic valvular regurgitation (Figure 3), systolic function was reduced and filling pressures were high. The left ventricle was hypertrophied in the study population. The measurements of both the interventricular septum and the posterior LV wall and mass were on average greater in the study group than that of the control group.

In addition to the above, right ventricular enlargement was noted in the patients with aortopathy when compared to controls, resulting in impaired early filling, and raised pulmonary artery systolic pressures implying long-standing disease and late presentation.

**Figure 4:**
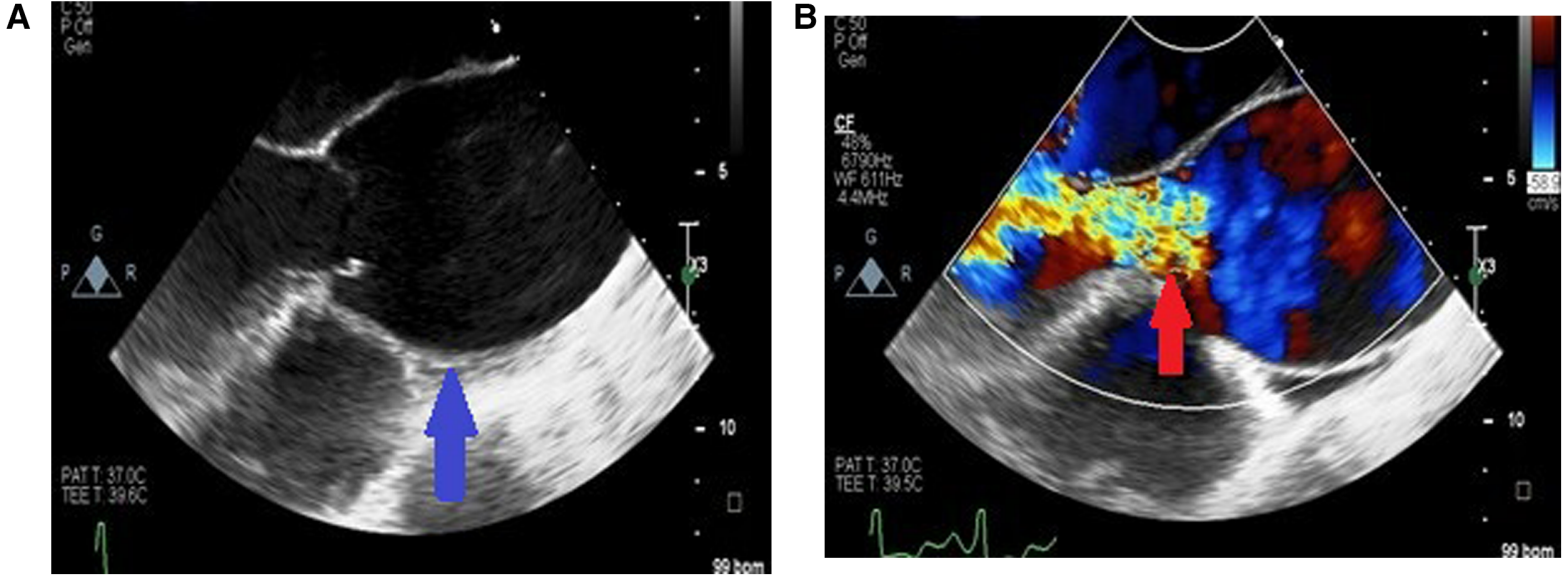
Long axis view of the aortic aneurysm in a patient with HIV showing enlargement from the root to the ascending aorta with effacement of sino tubular junction (blue arrow) (**A**) and malcoaptation of the leaflets with severe aortic regurgitation (red arrow) (**B**).

**Figure 5:**
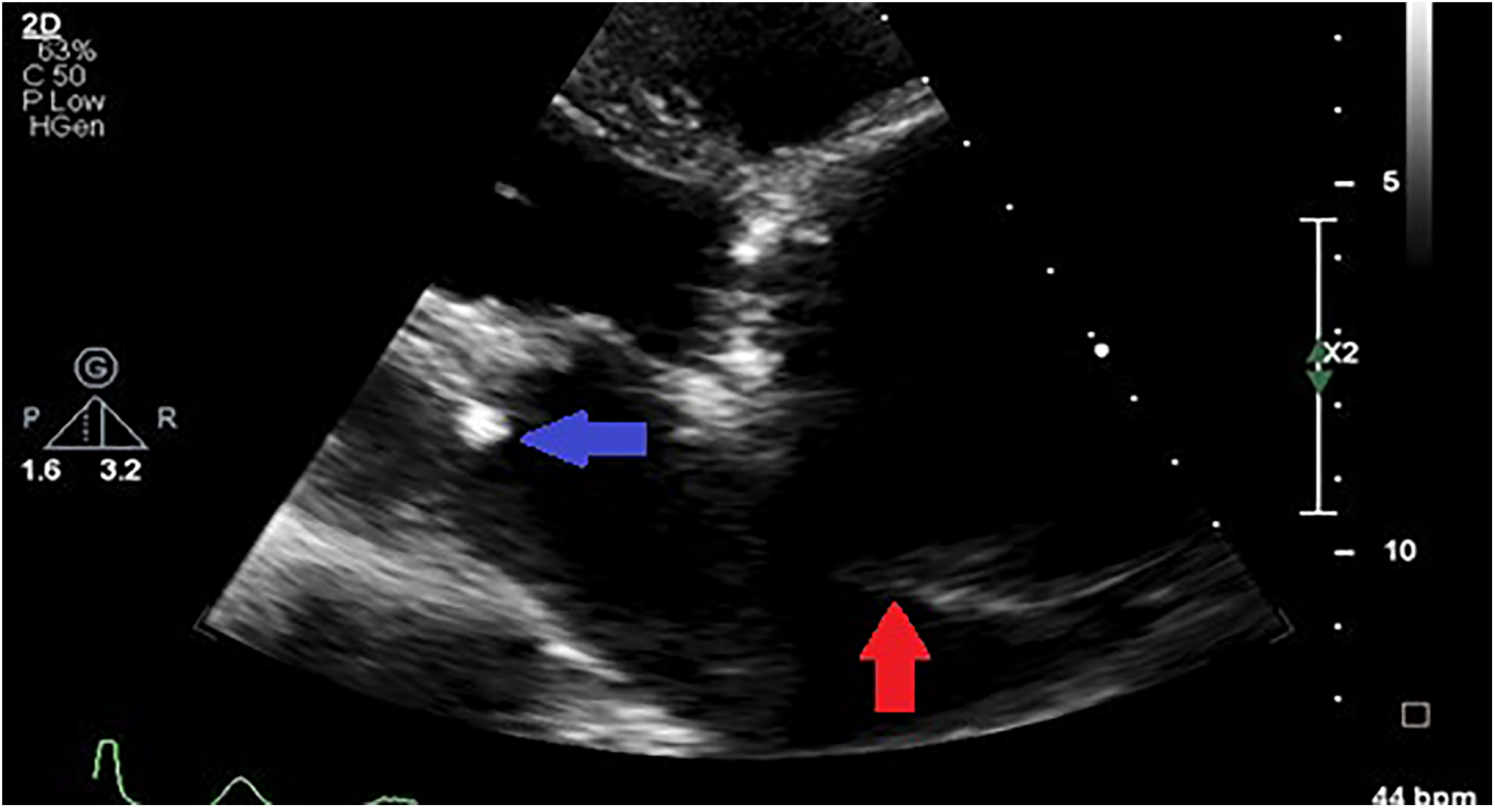
Parasternal off-axis view of the aorta in a hypertensive patient showing a markedly aneurysmal aorta compressing the left atrium (red arrow) and concurrent rheumatic mitral valve stenosis (blue arrow).

Most of the patients had functional aortic regurgitation (Table 2, Figures 2–4). The mean vena contracta (VC) width was 0.5 ± 0.2 cm, with an effective regurgitant orifice area of 0.2 (0.14–0.37) cm^2^ and an end-diastolic velocity of 18.2 (11.2–22.3) cm/s. In terms of the cases that had valvular structural abnormalities, there were two cases who had bicuspid valves (without aortic regurgitation or stenosis), one with degenerative aortic valve stenosis and one with mild rheumatic mitral valve stenosis. There was also a case of severe rheumatic mitral valve stenosis (Figure 5), one case with myxomatous mitral valve disease with mitral regurgitation and two patients who had mitral valve prolapse but no mitral regurgitation.

**Figure 6:**
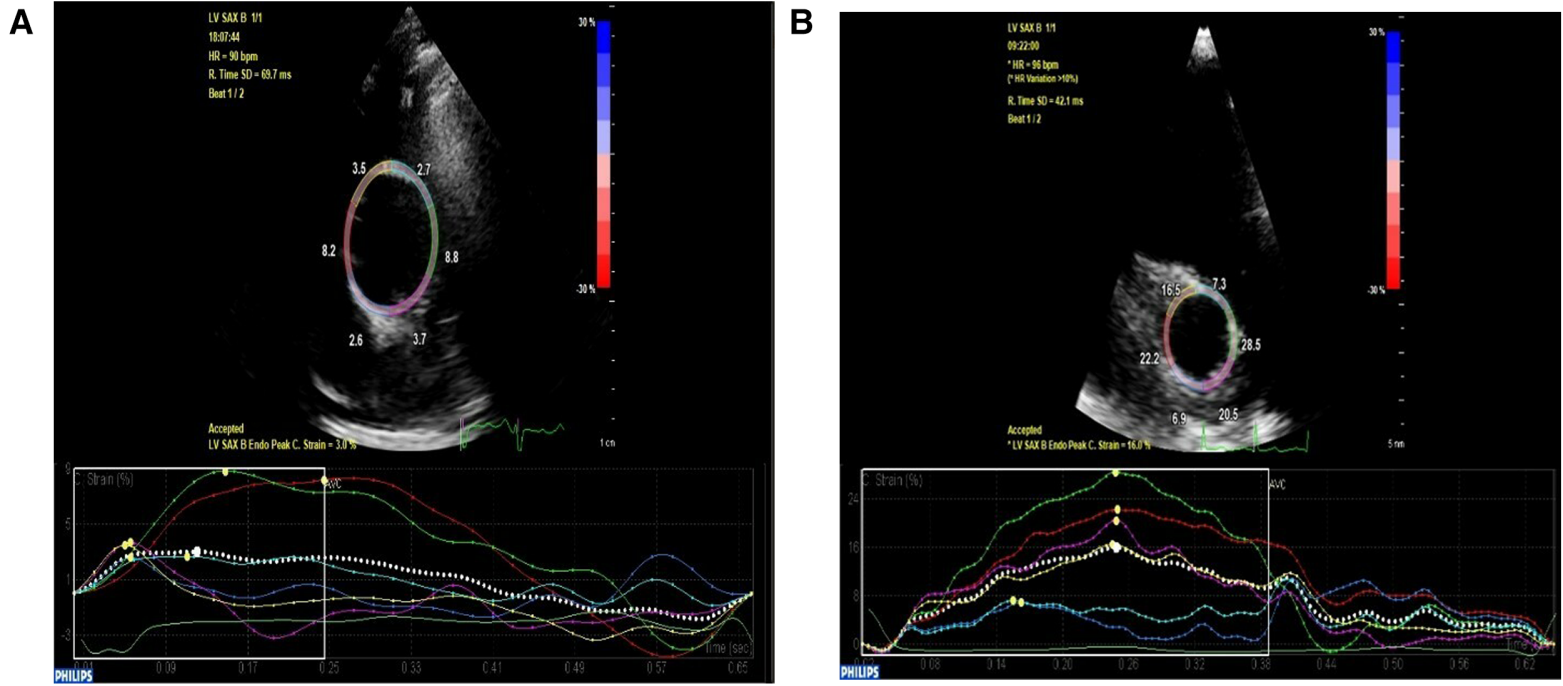
High parasternal short axis view of the ascending aorta depicting decreased circumferential strain at 3% in a patient with ascending aortic aneurysm (**A**) compared to age and gender-matched normal control with preserved circumferential strain at 16% (**B**).

The aortic circumferential strain was markedly reduced in the aortopathy group compared to controls (Figure 6). Further to this, the aortic stiffness index was higher in the aortic aneurysm group compared to controls, suggesting reduced compliance. Finally, left ventricular longitudinal and circumferential strain parameters were significantly reduced compared to controls.

Table 3 depicts the subgroup comparisons of patients with severe aortic regurgitation (AR) compared to patients with moderate and no or mild aortic regurgitation. Patients with severe aortic regurgitation had larger aortic diameters, most significant at the ascending aorta and sino-tubular junction level. There was no difference in CS between the three groups, however, the aortic stiffness index was higher in the severe aortic regurgitation group compared to mild or no AR group. There was no difference in the LV strain parameters between the three groups. Most of the patients with severe AR had HIV (57.9%) or hypertension (24.6%). Those with severe AR and connective tissue disease comprised 15.9% of the cases.

**Table 3:** Comparison of aortic parameters between severe and non-severe aortic regurgitation groups.

Parameter	Severe AR (*n* = 70)	Moderate AR (*n* = 36)	Mild and No AR (*n* = 33)	*P* value (ANOVA)
Age (years)	51.611 ± 14.89	52.47 ± 15.35	54.81 ± 16.42	0.610
Gender (M/F)	34/36	17/19	15/18	0.594
Body surface area (m^2^)	1.68 ± 0.21	1.76 ± 0.22	1.79 ± 0.26	0.057
Systolic blood pressure (mmHg)	135.34 ± 19.85	134.89 ± 20.27	143.59 ± 27.63	0.170
Diastolic blood pressure (mmHg)	66.39 ± 14.56	72.46 ± 13.46	86.81 ± 15.88^a^^,^c^^	<0.001
Aortic annulus (mm)	24.45 ± 5.22	23.29 ± 4.58	22.55 ± 4.12	0.152
Aortic annulus (mm/m^2^)	14.67 ± 3.14^c^	13.43 ± 2.89	12.87 ± 3.21	0.016
Aortic sinus (mm)	46.33 ± 9.35^c^	42.52 ± 8.33	41.00 ± 9.21	0.012
Aortic sinus (mm/m^2^)	27.89 ± 6.37^c^	24.37 ± 4.47^b^	23.39 ± 7.67	0.001
Sino-tubular junction (mm)	49.94 ± 10.38^c^	44.30 ± 7.39^b^	40.30 ± 9.74	<0.001
Sino-tubular junction (mm/m^2^)	30.08 ± 7.20^c^	25.32 ± 4.31^b^	23.18 ± 8.53	<0.001
Ascending aorta (mm)	24.45 ± 5.22^c^	23.29 ± 4.58	22.55 ± 4.12	<0.001
Ascending aorta (mm/m^2^)	14.67 ± 3.14^c^	13.43 ± 2.89^b^	12.87 ± 3.21	<0.001
LV Ejection fraction (%)	48.50 ± 13.36	48.00 ± 10.89	53.94 ± 12.19	0.087
LV Basal strain (%)	−14.42 ± 5.33	−14.31 ± 6.14	−12.34 ± 5.98	0.24
LV Apex strain (%)	−18.36 ± 8.47	−18.35 ± 7.00	−18.68 ± 11.17	0.985
LV Global longitudinal strain (%)	−13.78 ± 6.01	−13.70 ± 3.56	−13.77 ± 3.66	0.997
Circumferential strain of AAO (%)	4.82 ± 4.67	4.34 ± 2.46	3.78 ± 3.73	0.503
Aortic stiffness index (*β*_2_)	19.30 ± 25.40^c^	12.95 ± 12.82	8.75 ± 10.31^a^	<0.001

Data are presented as mean ± SD, median (IQR) or %. AAO, ascending aorta; AR, aortic regurgitation; LV, left ventricle.

Aortic CS and *β*_2_ was not feasible in 14 patients that presented with aortic dissection of the ascending aortic aneurysm due to intimal disruption.

^a^ Mild or no AR vs. moderate AR.

^b^ Moderate vs. severe AR.

^c^ Severe vs. mild or no AR.

### 3.3. Correlations between aortic regurgitation severity and aortic diameter

There was a positive correlation between AR severity parameters of vena contracta (VC) and end-diastolic velocity and aortic diameters. The most significant correlation was between VC and STJ diameter (*r* = 0.32, *P* = 0.003), and VC and AAO diameter (*r* = 0.34, *P* = 0.002). A significant correlation was also noted between AR end-diastolic velocity and STJ diameter (*r* = 0.29, *P* = 0.001), and AR end-diastolic velocity and AAO (*r* = 0.30, *P* < 0.001). Further to this, there was a positive correlation between sinus diameters and VC (*r* = 0.30, *P* = 0.004) and sinus diameter and end-diastolic velocity (*r* = 0.13, *P* = 0.03). However, there was no significant correlation noted between AR severity parameters and aortic annulus diameter (*P* > 0.05).

### 3.4. Blood biochemistry of the study patients

In terms of the blood biochemistry most (115) of the patients had borderline anaemia with a mean haemoglobin of 12.5 ± 2.6 g/dl. All of which had normal kidney function with urea of 6.5 ± 5.0 µmol/L and creatinine of 84 (68–107) µmol/L. The mean total cholesterol (56) was 3.9 (3.0–4.6) mmol/L. In group of HIV positive patients, the median CD4 count was (357–593 cells/µl). Nine had lower than detectable viral loads, while 27 patients had a median viral load of 1,020,000 (2,000,000–2,660,000) copies/ml.

### 3.5. Morbidity and mortality of the study patients

The morbidity due to heart failure and chronic aortic dissection was high in this study. All 14 patients in the chronic dissection group had uncontrolled hypertension with SBP ≥ 145 mmHg.

All patients were presented for surgery, except for 12 patients who either refused surgery or were considered too high of an intraoperative risk. Four patients demised while awaiting surgery, two patients refused surgery and then subsequently demised. A total of 41 patients (29.4%) ultimately underwent surgery. Five patients demised post operatively and one patient developed severe post-operative aortic regurgitation. The overall mortality was 7.9% (11/139). This is likely a significant underestimate as there was follow up of only 54% of the patients due to difficulties with contacting the patients. Of note, five patients with acute aortic dissection were excluded due to lack of data, three of these patients demised and two underwent successful surgery. The mortality for this subgroup was 40%.

## 4. Discussion

### 4.1. Distribution of TAA aneurysms by aetiology

Hypertension and HIV were the most common aetiologies in this study. In some cases, participants were noted to have multiple comorbidities making it challenging to discern the true aetiology of TAA aneurysm. In comparison to similar studies performed in African populations, Kitchen's 1980 study in Zimbabwe noted syphilis was the most common aetiology at the time, while syphilis was a suspected aetiology for TAA in only 4 of 139 patients in this study. In Kitchen's paper, hypertension was found to be less prevalent cause of TAAs. However, it was predicted that hypertension would become a predominant cause for TAAs due to a shift towards a westernized lifestyle and diet in African populations (7). This forecast has proved true in the results from the current study as well as the studies by Ogeng'o et al. in Kenya (4) and Mvondo et al. (25) and Antunes et al. in South Africa (26) wherein hypertension were found to be the predominant aetiology of TAAs.

To our knowledge this is the first study performed in an African population to attribute HIV as a cause for TAAs. In all four similar studies performed on the continent, HIV has not been specifically mentioned as a cause for TAAs. This is likely due to studies being done in the pre-HIV era and due to the higher rate of HIV in South Africa contributing to the higher burden of TAA aneurysms when compared to other African countries.

### 4.2. Clinical characteristics of patients with TAA aneurysms

Regarding sex and age, in this study the predominant sex was female and the mean age of patients with TAA aneurysms is 50 years old, which correlates with findings by Kitchen and Antunes et al. By contrast the study by Ogeng'o et al. had an older (56 years on average) female predominant population, while Mvondo et al., had a younger mean age of 43 years who were predominantly male.

Dyspnoea due to heart failure as well as aortic dissection were a frequent causes of index presentation in this study. Ten percent of patients presented with aortic dissection and 70% presented in heart failure. This is fewer compared to the study by Mvondo et al., where 30% of patients presented with dissection and 90% of patients who presented with dyspnoea. The overall mortality in this study was lower compared to other centers in Africa (4, 25). However, it is likely to be underestimated as only a third of the patients ultimately received surgery. This was due to limited expertise, selection bias, poor follow-up data and scarcity of resources. High mortality of acute aortic dissection was consistent with the literature (27).

A high prevalence of hypertension (67.3%–76.6%) has been reported in patients with aortic dissection (28, 29). This was true for the patients in this study where, majority of the patients were hypertensive suggesting that this is a major risk factor for aortic dissection in this study population. In this study, the patients with aortic dissection also had larger aortic diameters. This is likely due to hypertension playing a role in increasing wall stress, predisposing the aortic wall to dilatation and ultimately dissection.

A meta-analysis showed that the risk of aortic dissection increased at a systolic blood pressure >132 mmHg and diastolic blood pressure >75 mmHg, highlighting the importance of stringent blood pressure control. Therefore, it is suggested that to reduce risk of aortic dissection, the mean blood pressure goals should be even lower than the threshold for hypertension (30).

### 4.3. Impact of aortic regurgitation on aorta size, aortic stiffness index and LV mechanics in TAA aneurysms

In the current study, half of the patients with aortic aneurysms had severe AR. AR has been shown to have a negative impact on the aortic wall secondary to increased shear wall stress (31). This is caused by the remodeling of the medial layer and apoptosis of the cells in the media due to marked medial remodeling and medial cell apoptosis, possibly related to dysregulated endothelial nitric oxide synthase (eNOS) signaling in the endothelium. Thus, a vicious cycle may be created whereby aneurysmal dilation results in severe aortic regurgitation which in turn results in further enlargement of the aorta.

A subgroup analyses of aortic aneurysm patients with severe AR, moderate AR and mild AR was performed. Despite no difference in systolic blood pressure and ejection fraction compared to non-severe AR group and a lower diastolic blood pressure, patients with severe AR had larger aortic diameters, with the most significant difference noted at the level of the sino-tubular junction (STJ) and ascending aorta. Furthermore, there was a statistically significant positive correlation between the parameters of AR severity and aortic diameters of the root and ascending aorta. Therefore, there is a complex interaction between AR severity and aorta dilation. From our findings it seems that all components of the aortic root likely contribute to aortic regurgitation, but the STJ and ascending aorta play a more dominant role. This correlates with the study by Wenzel et al. who also concluded that the STJ diameter had the greatest association with aortic regurgitation severity in an aging population (32). Hypertension is associated with dilation of STJ due to disease of the media (33) and this is a likely mechanism of AR in hypertensive patients with normal leaflet morphology but mal-coaptation. However, in this study it was noted that most patients with severe AR were HIV reactive and we speculate that HIV valvulitis, as an extension of aortitis (34), also contributed to valvular regurgitation in this subgroup of patients.

Advanced imaging of the aortic circumferential strain (CS) and calculation of the aortic stiffness index revealed stiffer aortas in the patients with aortic aneurysms, portending an increased risk of further dilation and dissection (13). Further, we noted that compared to non-severe AR groups in this study, severe AR was also associated with greater aortic stiffness, further adding to the risk of dissection and likely signaling a poorer prognosis for this subgroup of patients with aortic aneurysms. Poor distensibility of the aorta has been correlated with faster hemodynamic deterioration and rapid progression of disease in patients with aortic regurgitation (35).

All left ventricular longitudinal and circumferential strain parameters were significantly reduced compared to controls. Except for the LV mass which was greater in study patients, likely secondary to hypertension and/or severe aortic regurgitation causing an increased LV preload and afterload (35). No difference was noted between LVEF and strain parameters according to AR severity and this could be partly explained by the influence of co-morbidities on reduced LV function in all three groups independent of AR.

Initially, as the LV longitudinal function declines the circumferential function increases to maintain ventricular contractility and cardiac output (36). As the disease progresses the circumferential compensation likely declines and the ventricle fails. The decline in both longitudinal and circumferential function of the patients' ventricles in this study implies long-standing disease secondary to delayed diagnosis. The former statement is also supported by the markedly increased left ventricle end-diastolic and end systolic volumes, LV mass, as well as the impaired diastolic relaxation and increased left atrial volumes of the study patients.

### 4.4. Role of echocardiography in TAA aneurysms

Echocardiography, especially Speckle-Tracking analysis, has been shown to be an accessible and useful tool for the detection of TAA aneurysms and measurement of circumferential vascular mechanics (12) in a resource-limited setting where cardiac computed tomography (CT) services are overburdened with resultant delayed diagnosis (37). Guideline recommendations for screening of thoracic ascending aortic aneurysms are scarce compared to those for abdominal aortic aneurysms. Currently, guidelines suggest screening older patients and those with risk factors for aneurysm formation (38). Several countries have attempted to implement surveillance programs for aortic aneurysm detection; however, resource limitations have made these programs difficult to replicate (39). However, this retrospective data is hypothesis generating and future studies would be needed to define the role of echocardiography in screening TAA.

## 5. Conclusion

This is the first study from Africa detailing the differences in clinical and echocardiographic characteristics in those with thoracic ascending aortic aneurysms compared to controls. Thoracic ascending aortic aneurysms were mostly present in young black African females and the main aetiologies were hypertension and HIV. This study reveals a high burden of ascending aortic aneurysms in the study population, which largely develop undetected and ultimately present late with advanced heart failure or aortic dissection. Delayed presentation of TAA aneurysms results in considerable morbidity and mortality and highlights the need for (i) prevention and control of modifiable risk factors for aortic aneurysms such as hypertension and HIV and (ii) screening programs and establishment of centers of excellence for effective management of aortic aneurysms in Africa. Echocardiography has been presented as a useful low-cost tool for the detection of aneurysms and potential risk stratification using advanced strain imaging, however further studies are needed to define its role in screening in this population.

## 6. Limitations

In terms of the limitations of this study, the study comprised a predominantly African population and therefore limiting the applicability to other populations. Morbidity and mortality data could not be fully accessed due to poor patient follow-up. Furthermore, interobserver variability may have affected the measurement of echocardiographic parameters, however, standard deviations of measurements were small and like those reported in other studies and so interobserver variability influence was negligible. The absolute values of aorta CS are subject to inter-vendor differences.

## Data Availability

The raw data supporting the conclusions of this article will be made available by the authors, without undue reservation.
